# Data-Driven Slip Prediction in Web Processing Machines Using Virtual Sensors and Ensemble Machine Learning

**DOI:** 10.3390/s26092878

**Published:** 2026-05-05

**Authors:** Colin Soete, Jonas Van Der Donckt, Nathan Vandemoortele, Jasper De Viaene, Jeroen De Maeyer, Sofie Van Hoecke

**Affiliations:** 1 IDLab, Department of Electronics and Information Systems, Ghent University—Imec, Technologiepark-Zwijnaarde 122, 9052 Zwijnaarde, Belgium; jonvdrdo.vanderdonckt@ugent.be (J.V.D.D.); nathan.vandemoortele@ugent.be (N.V.); sofie.vanhoecke@ugent.be (S.V.H.); 2 DySC, Department of Electromechanical, Systems and Metal Engineering, Ghent University—Flanders Make, Sint-Martens-Latemlaan 2B, 8500 Kortrijk, Belgium; jasper.deviaene@ugent.be (J.D.V.); jeroen.demaeyer@ugent.be (J.D.M.)

**Keywords:** virtual sensor, web winding machine, slip prediction, roll-to-roll manufacturing, predictive maintenance, ensemble learning

## Abstract

In roll-to-roll (R2R) web processing systems, traction rollers impose precise velocity profiles on the moving web. Ideally, the web follows this trajectory without deviation, but slip can occur during rapid acceleration or deceleration, leading to tension loss and degraded product quality. Although slip can be detected directly using high-resolution encoders that track the actual web speed, such sensors are expensive and require machine downtime for installation, making them impractical for large-scale industrial deployment. To overcome this limitation, we developed a virtual slip sensor that estimates slip using existing machine signals only. A temporary encoder was used to collect ground-truth data, enabling the training of predictive models that eliminate the need for a permanent physical sensor. The proposed system employs an ensemble modeling approach: a CatBoost model captures low-slip behavior where data is abundant, while a linear model extrapolates to high-slip, out-of-distribution conditions. Targeted feature engineering ensures generalization across varying ramp times and web speeds. Despite being trained primarily on data containing limited slip, the models successfully generalized to scenarios with severe slip, demonstrating robust predictive performance. The ensemble reduces the regular CatBoost model’s MSE at 60 m/min by approximately 54% in the speed-based evaluation and by approximately 68% in the quantile-based evaluation while maintaining comparable performance in the low-speed regimes. The resulting virtual sensor enables continuous real-time slip monitoring, providing operators with timely insights to prevent quality degradation and operate at higher acceleration profiles to increase throughput, even on machines that have not previously experienced extreme slip.

## 1. Introduction

Modern manufacturing industries increasingly rely on automation and data-driven methods to improve efficiency, reduce waste, and maintain consistent product quality [1,2]. These developments are particularly evident in roll-to-roll (R2R) processing systems, where a flexible web is unwound, guided through multiple processing stations, and rewound in a continuous manner [3]. R2R manufacturing is widely used in printing, packaging, battery production, and flexible electronics, where even small disturbances in web motion can propagate through the line and degrade product quality [4,5].

Winding systems within R2R processes can be broadly categorized into one-dimensional (1D) and two-dimensional (2D) configurations. While 1D systems focus on longitudinal motion and tension control, 2D systems add lateral actuation for precise path and edge alignment. The 2D web winding machine studied in this work is a complex mechatronic multi-motion system equipped with sensors and actuators that jointly regulate web speed, tension, and position. Maintaining precise tension is essential for preventing wrinkles, tearing, misalignment, and other defects that compromise product quality [6,7]. However, despite advanced control strategies, certain physical phenomena, most notably slip between the web and traction rollers, introduce nonlinearities that are difficult to capture with conventional PID-based or model-based control approaches [8].

In roll-to-roll web processing systems, traction rollers play a fundamental role in imposing precise velocity profiles onto the web material. These rollers, typically coated and driven by electric motors, serve as master actuators that carry the transport across the entire process line [9]. To prevent deformation, web-specific velocity trajectories must be carefully designed and executed. Under ideal conditions, the web adheres perfectly to the imposed velocity profile. However, slippage between the traction roller and the web can occur, particularly during dynamic transitions such as start–stop sequences or rapid accelerations [10]. This slippage leads to tension drops, process instability, and degradation in the quality of the final product winding.

Many R2R subprocesses, such as printing, gluing, heating, and stamping, require operation at reduced speeds due to the nature of the process or material constraints. Consequently, these subprocesses often run slower than the unwinding group. To counteract these speed differences while maintaining continuous web flow, accumulator systems are employed. These systems buffer the web material and enable discrete start–stop operations, resulting in frequent acceleration and deceleration phases over short time intervals. These dynamic transitions significantly increase the risk of slippage due to the demanding mechanical conditions.

Slip can be measured directly using position sensors that track the actual web speed, typically by monitoring the rotation of a free roller. However, the sensors commonly installed in industrial machines are typically low-cost. These low-resolution sensors are generally sufficient for measuring the cumulative web length but lack the precision required to detect slippage during short, high-acceleration events. Accurate slip detection under such dynamic conditions demands high-resolution sensors, which are significantly more expensive [11]. Therefore, these sensors are impractical for widespread deployment in industrial environments.

In practice, slippage can be mitigated by dynamically limiting the velocity trajectories. Reducing acceleration reduces the likelihood of slip but also extends cycle times, which is undesirable in high-throughput industrial environments. Another approach is to increase the tension of the web, thus enhancing the normal force at the roller interface and delaying the beginning of slippage. However, excessive tension can deform sensitive materials and is generally avoided due to material limitations and the increased energy consumption associated with higher torque demands.

Beyond mechanical factors, selecting the appropriate roller coating for a given web material is still largely a manual, trial-and-error process. Operators frequently compensate for uncertain frictional conditions by conservatively tuning tension parameters or limiting machine speeds, which can be suboptimal in high-mix production environments requiring rapid adaptation [12].

An additional challenge for slip mitigation in industrial R2R systems is the need to generalize beyond the operating regimes captured during data collection. In practice, it is infeasible to experimentally cover all combinations of web materials, coatings, machine configurations, and speed profiles within the limited commissioning time available. Moreover, potentially harmful regimes, such as extreme accelerations or high-speed operation, are often underrepresented [13] or deliberately avoided during experiments due to safety and quality concerns. As a result, severe slip events may occur under conditions that are not present in the training data of a data-driven slip prediction pipeline. This makes generalization to out-of-distribution operating regimes a critical requirement for slip prediction models intended for safe and reliable industrial deployment.

To address these limitations, we propose a data-driven virtual sensor to detect and predict slip in 2D R2R web winding machines. Using existing onboard sensors, we generate labels from a temporary high-resolution encoder and train models capable of estimating slip without requiring permanent hardware modifications. This approach provides detailed insight into the operational boundaries of the system and enables predictive detection of slip events across a range of operating conditions.

This paper presents a novel data-driven slip prediction framework designed for practical deployment in industrial winding machines. Our key contributions are as follows.

- Encoder-free virtual slip detection: We propose a virtual slip sensor that eliminates the need for a dedicated encoder sensor by learning correlations between the web and roller speeds using existing onboard sensors only. A temporary encoder is used solely for label collection during training, which enables real-time slip estimation without permanent hardware modifications.
- Feature engineering for slip prediction: We develop a comprehensive set of time-series features to model the onset and severity of slip. These features capture dynamic interactions among roller speeds, torque, and tension under varying operating conditions.
- Regime-aware ensemble architecture: We propose an ensemble that purposefully combines linear and non-linear models, exploiting their complementary strengths across the different slip regimes in which they perform best. This design directly addresses the challenge of reliable slip prediction under severely imbalanced data conditions, where the most critical operating regimes are underrepresented in the training data.
- Out-of-distribution evaluation in harmful operating regimes: We explicitly evaluate model performance under out-of-distribution conditions characterized by severe slip events that are deliberately avoided during normal data collection. To the best of our knowledge, this is the first work to investigate such evaluation in the context of R2R web processing machines. This analysis provides insight into the model’s ability to generalize beyond nominal operating regimes for safe industrial deployment.
- Public dataset for slip prediction: We release a public dataset containing slip scenarios across diverse machine states. A shared experimental benchmark is currently lacking, and its availability would promote reproducibility, transparent comparison, and community-driven innovation in slip detection research.

The remainder of this manuscript is organized as follows. Section 2 reviews related work and highlights the limitations of existing approaches. Section 3 presents the use case for our study and details the dataset specifications. In Section 4, we describe the methodology for data processing and model training. Section 5 reports the results and discusses key findings. Section 6 discusses the broader understanding of practical use cases and its limitations. Finally, Section 7 summarizes the conclusions and outlines potential directions for future work.

## 2. Related Work

Martin et al. [3] reviewed prevalent techniques in roll-to-roll manufacturing, highlighting the challenges in maintaining product quality and process reliability. Winding quality faults are well-documented in the literature. For instance, improper roll alignment can result in telescoped, dished, tight or loose rolls [14,15]. Mechanical issues such as belt tension problems, belt or gear damage, and bearing failures can also further compromise system performance [16].

Slip between the web and traction rollers is a critical phenomenon in R2R systems, as it directly affects tension control, alignment, and ultimately product quality. Several studies have addressed slip from both mechanical and modeling perspectives. Hashimoto et al. [17] propose an approach to manage tension in the wind-up roll to prevent wrinkling, creep, and slippage. They suggest an optimization technique to manage this in-roll stress based on mathematical techniques such as cubic splines for calculating the tension distribution. Wu et al. [18] analyze the cause of axial slip by studying the force of the center-wound roll. They used mathematical modeling to analyze the mechanical relationship between the layers of the center winding roller and established a correlation model between winding tension and axial slip.

Other studies have focused on sensorless estimation of tension and slip to avoid costly equipment. For example, Hwang et al. [19] designed a Kalman-filter-based tension control strategy combined with a disturbance observer, capable of compensating for slip and inertia variations without additional sensors. Similarly, Huang et al. [20] proposed an $H_{\infty }$-based sensorless estimation framework for R2R processes that accounts for friction and external disturbances. These works highlight the potential of observer-based methods to maintain performance under uncertain slip conditions, although they remain rooted in model-based control.

AI-driven virtual sensing and digital twin methodologies have emerged as complementary approaches in roll-to-roll systems. Gafurov et al. [21] demonstrated web tension and speed reconstruction in an R2R digital twin using AI-based modeling. They show that statistical and frequency-domain features can be used to predict tension without additional sensing hardware. However, the generalization of such models to out-of-distribution operating conditions, particularly those associated with potentially harmful or extreme system behavior, remains unaddressed. Evaluating model performance under these conditions is crucial to understanding reliability and safety margins in industrial deployment. This out-of-distribution generalization challenge is a recurring limitation across data-driven industrial monitoring approaches. Labeled fault data or extreme operating conditions are scarce and models trained on nominal regimes often fail to extrapolate reliably [22,23].

Similar digital twin approaches have been explored for accumulator dynamics [24] and fault diagnostics in R2R systems [25], further underlining the relevance of data-driven monitoring frameworks in continuous web processing.

While these studies provide valuable insights, they generally rely on either precise mechanical models or simulation-based digital twins, which often require significant calibration and engineering effort. In contrast, data-driven virtual sensors provide a promising alternative by estimating slip using existing onboard measurements. Ponsart et al. [14] demonstrated the use of virtual sensing to detect sensor faults in web winding systems, highlighting the feasibility of such data-driven fault estimation. More broadly, soft sensing or virtual sensing has emerged as a practical paradigm across manufacturing applications where physical sensors are costly, fragile, or difficult to install. In turn, virtual sensing enables the estimation of process variables from existing instrumentation [26].

Despite these advances, there remains a lack of publicly available datasets containing comprehensive sensor measurements of web winding systems. This absence limits reproducibility and the development of generalized, data-driven models for slip prediction and related fault detection. Furthermore, while data-driven approaches for estimating web tension and roller speed have been previously investigated in web processing systems, existing studies primarily focus on stable operating conditions. To the best of our knowledge, no prior work has proposed a fully data-driven virtual slip sensor operating exclusively on existing onboard signals in R2R web processing machines. Similarly, no prior work explicitly evaluated predictive performance under out-of-distribution, high-slip operating regimes in this context. Addressing these gaps is the primary motivation for the work presented in this paper. To address the generalization challenge, we incorporate a regime-aware ensemble strategy that combines a gradient boosting model with a linear extrapolation model. This architecture exploits the models’ complementary strengths across the different slip regimes in which they perform best. The design is motivated by the established robustness of ensemble methods in industrial condition monitoring [27].

## 3. Use Case

**Figure 1:**
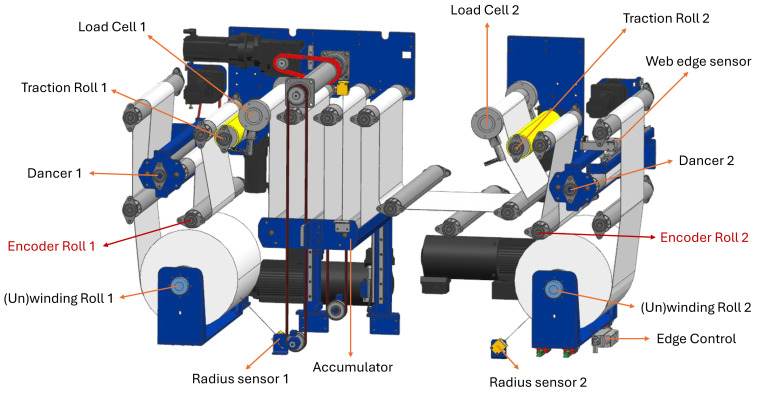
Schematic of the experimental web processing system. Principal mechanical components include the winding and traction rollers, dancers, encoders, and an accumulator.

A physical web processing unit is designed and built with actuators and sensors to control the web speed and tension, while its digital twin model can be found in Figure 1. Maintaining consistent web tension throughout the processing line, even under varying web speeds, is critical to ensure high product quality [28,29]. The primary purpose of the unit is to emulate processes such as cutting, printing, and stamping on the web without interruption. Achieving this requires precise control to minimize defects and maintain process robustness.

The web winding machine consists of several key components, illustrated in Figure 1. The **winding roller** ((Un)winding Roll 1, (Un)winding Roll 2) unwinds the roll on the left or winds it on the right, with the web direction being adjustable. **Traction rollers** (Traction Roll 1, Traction Roll 2) are typically positioned every 5 m to maintain tension along the web, driving the material and controlling its speed. The wrapping angle of the web around each traction roller can be modified by adjusting one of the support rolls to one of five discrete positions.

The **accumulator** buffers the web by moving vertically, allowing the material to momentarily stop for processes such as printing. The **dancer** maintains tension by rotating and buffering additional material, in order to remain level (0°) through an electrically controlled spindle. A **load cell** measures web tension, functioning like a scale and recording force along three axes. Detailed specifications of the sensors and actuators are provided in Table A3, while motor specifications are listed in Table A4.

The web processing unit has the following dimensions: 2.1 m in length, 1.4 m in width, and 2.2 m in height. Key operational limits include a maximum web speed of 120 m/min, a maximum web tension of 325 N, and a maximum web width of 60 cm.

The web material selected for our use case is a waterproofing membrane called Kerdi, with specifications provided in Table 1. Kerdi is a non-woven polyethylene fabric (manufactured by Schluter) used for waterproofing tiles. It is elastic and features a woven pattern that deforms under tension. Nevertheless, the machine is equipped with all the essential components required to handle and process a variety of web materials.

**Figure 2:**
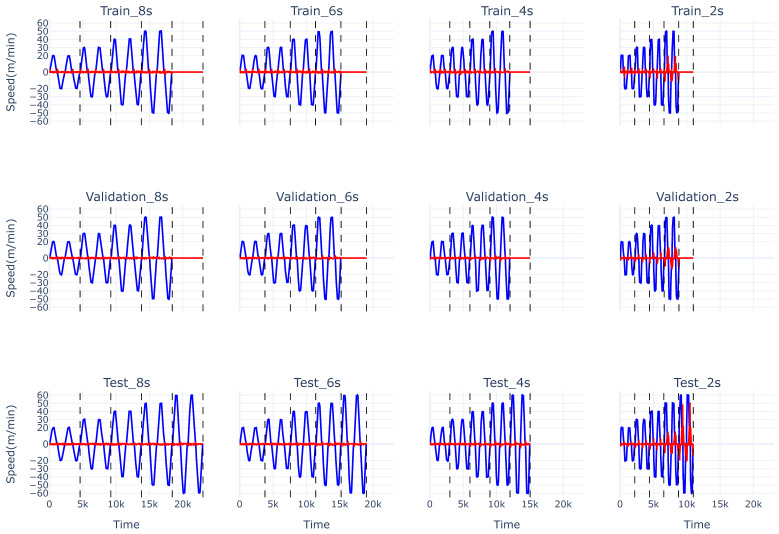
The various operational configurations of our use case with the web speed depicted in blue and the slip label values illustrated in red. The training, validation, and test sets are shown from the top to bottom row respectively. The ramp time gets progressively shortened from left to right for each set. The vertical dashed lines divide the distinct web speeds regimes found in Table 2.

Data from the web winding machine is acquired from Siemens S1500 PLC controllers at a sampling rate of 50 Hz. The dataset consists of multiple test runs in which three variables are independently varied: ramp time (2 s, 4 s, 6 s, 8 s), web speed (20 m/min, 30 m/min, 40 m/min, 50 m/min, 60 m/min), and web direction. A short pause of 10 s is introduced between changes in each variable to allow the machine to return to its initial state. Once the target web speed is reached, it is maintained for 10 s before decelerating back to a standstill. The direction of the web is continuously alternated, and each configuration is repeated twice. Web speeds of 0 m/min and 10 m/min were not included as these contained minimal to no slip, so it was trivial to predict slip in these regimes. The experimental protocol is illustrated in Figure 2.

Slip (*S*) may occur when the ramp time is too short and/or the target web speed is too high, resulting in steep acceleration and deceleration slopes in the web speed profile. While slip can occur between various components and rollers along the web path, this study focuses specifically on the slip between the unwinding roll and the first traction roll, as this location directly influences material tension and downstream web stability. Slip can be estimated in multiple ways, for example, by comparing the traction roller speed to the linear speed of the (un)winding roll:

(1) $$S_{simple}=v_{\mathrm{traction}\text{\_}\mathrm{roll}1}-n_{(\mathrm{un})\mathrm{winding}\text{\_}\mathrm{roll}1}\cdot 2\pi \cdot r_{(\mathrm{un})\mathrm{winding}\text{\_}\mathrm{roll}1}$$

where $v_{\mathrm{traction}\text{\_}\mathrm{roll}1}$ is the linear speed of traction roll 1 (m/min), $n_{(\mathrm{un})\mathrm{winding}\text{\_}\mathrm{roll}1}$ is the rotational speed of unwinding roll 1 (rpm), and $r_{(\mathrm{un})\mathrm{winding}\text{\_}\mathrm{roll}1}$ is the current radius of unwinding roll 1 (m).

In our case, the slip label is determined using an encoder, as it provides greater accuracy:

(2) $$S_{actual}=-n_{\mathrm{encoder}\text{\_}\mathrm{roll}1}\cdot 2\pi \cdot r_{\mathrm{encoder}\text{\_}\mathrm{roll}1}-v_{\mathrm{traction}\text{\_}\mathrm{roll}1}$$

where $n_{\mathrm{encoder}\text{\_}\mathrm{roll}1}$ is the rotational speed measured by encoder roll 1 (rpm), $r_{\mathrm{encoder}\text{\_}\mathrm{roll}1}$ is the fixed radius of encoder roll 1 (0.037 m), and $v_{\mathrm{traction}\text{\_}\mathrm{roll}1}$ is the linear speed of traction roll 1 (m/min). The negative sign on the encoder term accounts for the opposing rotational direction of the encoder roll relative to the traction roll.

The machine provides several signals associated with the rollers, traction units, dancer, and accumulator. The position signal typically represents vertical movement (height), except for the dancer, where it corresponds to rotational motion (angle). The torque signal reflects the rotational force applied by the motor, measured in Nm. Speed is recorded as an angular or linear velocity in m/min, while the setpoint indicates the target speed of the motor controller, also in m/min.

In addition, two sensors are used to measure the roll radius: RadiusRough and RadiusFine. Both sensors measure the same quantity. However, RadiusRough is relatively noisy, while RadiusFine provides higher accuracy and is therefore preferred for conversions. Finally, an Encoder Roll 1 records the actual ground truth web speed, measured as linear velocity in m/min after conversion. On Figure 1, these encoder sensors are highlighted in red.

**Table 2:** Web speed and ramp time configurations for all data splits. “All” signifies the three distinct parts, namely training, validation and test set. Note that the test set also contains an out-of-distribution web speed (60 m/min) across all ramp times.

		Ramp Time (s)			
		8	6	4	2
Web speed (m/min)	60	Test	Test	Test	Test
	50	All	All	All	All
	40	All	All	All	All
	30	All	All	All	All
	20	All	All	All	All
	−20	All	All	All	All
	−30	All	All	All	All
	−40	All	All	All	All
	−50	All	All	All	All
	−60	Test	Test	Test	Test

## 4. Methodology

**Figure 3:**
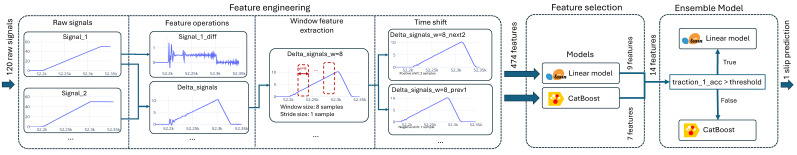
Schematic overview of the data-driven pipeline for slip prediction.

The general overview of the methodology section can be found in Figure 3. The methodology is divided into three primary stages: feature engineering of raw sensor signals, greedy forward feature selection, and the development of a hybrid ensemble model integrating linear regression and CatBoost to optimize performance across varying operational regimes.

**Figure 4:**
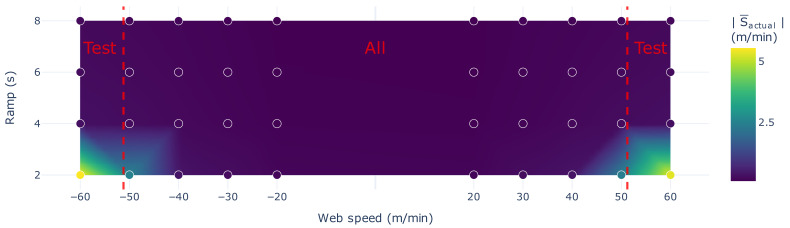
Contour plot illustrating the mean absolute slip values across the experimental parameter space. The horizontal axis represents the steady-state web speed in m/min, while the vertical axis denotes the ramp times in seconds.

The data is split into three parts; a training part, a validation part, and a test part. All parts were recorded consecutively during the same day. The training and validation sets share identical web speed and ramp time configurations. This results in a similar number of data points, which facilitates reproducibility of model evaluation. However, the test set progressively includes more instances of elevated slip. As shown in Table 2, the test set is the only split that contains web speeds of 60 and −60 m/min, where positive values indicate that the (un)winding roll 1 is unwinding the web material, while negative values correspond to a reversal of web direction in which roll 1 is winding the web material. Furthermore, Figure 4 shows the mean absolute slip value for each combination of ramp time and web speed. Discrete markers indicate the specific operational configurations evaluated, with the color gradient interpolated to show the transition between stable (un)winding and high-slip regimes. The figure also distinguishes between the full dataset and the test set, highlighting that the more extreme web speed conditions in the test set are associated with elevated mean absolute slip values. By training and validating on data with relatively limited slip and testing on scenarios with potentially critical slip, the model can provide valuable insights into the system’s operational boundaries without inducing damaging conditions to the setup. Therefore, this design allows us to assess the model’s ability to generalize to out-of-distribution operating regimes that differ substantially from those observed during training.

### 4.1. Feature Engineering

The raw data serves as an input to the feature engineering part, as displayed in the leftmost block of Figure 3. The different feature manipulation methods that are discussed in this section can also be visually seen on this schematic. We focus particularly on raw data originating from the sensors surrounding the encoder 1 roll (see Figure 1), as we hypothesize that these signals are most strongly correlated with the encoder roll measurements. These neighbouring signals include data originating from traction 1 roll, dancer 1 and (un)winding roll 1. Additional features are constructed for each split of the dataset, including shifted differences between elements in a vector, deltas (Δ) between signals, and shifted differences of these deltas. For instance, the “diff” function in the pandas library (https://pandas.pydata.org/) computes the difference between consecutive elements in a series, such as the motor speed of the traction roll, which can provide an estimate of acceleration given our fixed sampling rate. Deltas between signals can be calculated by, e.g., subtracting the motor speed of traction roll 1 from that of traction roll 2. Logical differences and deltas are generated where relevant, reflecting the objective of predicting slip at that particular location.

A sliding window approach is employed to perform feature extraction from both the original and constructed signals for modeling purposes. Multiple standard statistical features are extracted, including the mean and standard deviation over different window sizes. In addition, the first and last values, as well as the differences in the signals, are computed using a window size of two samples. A stride of one sample is applied to each calculated window size. This process can be efficiently implemented using the tsflex library [30]. The complete procedure for constructing new signals and extracting features is available at https://github.com/predict-idlab (accessed on 27 February 2026).

After initial feature extraction, temporal shifts (lags) are applied to the features to account for inherent time delays and phase shifts between different components of the web processing system, such as actuators, rollers, and sensors. Due to transport delays and controller dynamics, changes in one subsystem may influence downstream measurements only after a finite time. To capture these delayed interactions, features extracted with window sizes of f and 8 samples are shifted with a maximum of two forward and two backward shifts. Features extracted with a window size of 2 are shifted using increments of 2, 4, 8, and up to 16 samples. These shifted features are then combined with the previously constructed feature subset to form the final feature set for modeling. The eventual shape can be seen on Figure 3 as an output from the feature engineering block and contains 474 features. The constructed feature subset is now ready to serve as input for our predictive models.

### 4.2. Feature and Model Selection

A linear model and a CatBoost model were separately trained on the full training set and subsequently evaluated on the training, validation, and test sets for both low-speed and high-speed segments. Before modeling, feature selection was performed because the original feature dimension of 474 could lead to issues related to the curse of dimensionality. To address this, the feature space was reduced using a simple forward feature selection method. This procedure was executed independently for both the linear and CatBoost models, each trained on the training partition of the data and is shown in the middle block of Figure 3.

The evaluation metric selected for this study is the Mean Squared Error (MSE), where lower values indicate better predictive performance. MSE measures the squared difference between predicted and actual slip values, thereby heavily penalizing larger errors. This characteristic is particularly important because the slip values under extreme operating conditions exhibit an approximately exponential behavior. This exponential slip behavior can be seen on Figure 2 when increasing the web speed and/or decreasing the ramp time. Using MSE as the evaluation metric emphasizes these larger slip errors, which is critical to prevent irreversible damage to the system.

Feature engineering was initially designed with linear models in mind, as these models enable direct interpretability of sensor correlations with slip. By examining the learned coefficients of a linear model, we can infer which sensor signals contribute the most to the prediction of slip, thereby providing valuable physical insights into the process. The linear modeling pipeline consists of a StandardScaler followed by a Lasso regression model from scikit-learn (https://scikit-learn.org/), which also performs feature selection through regularization. Other linear models, such as SGDRegressor, Ridge, SVR, were also evaluated, and Lasso proved to be the best performing model due to its ability to penalize high-slip regions. The implementation for the other linear models can be found in the accompanying repository.

To complement this interpretable linear approach, a CatBoost [31] model was introduced to capture nonlinear relations that may exist among sensors. CatBoost is a gradient boosting algorithm based on ensembles of decision trees, where the model is built sequentially by iteratively adding trees that are trained to reduce the residual errors of the preceding ensemble. The final prediction is obtained as the weighted sum of all trees. This model is particularly effective for modeling complex dependencies in multivariate industrial data. Our hypothesis is that CatBoost generalizes well in low-slip regions where abundant data allows the model to learn intricate relationships, while linear models provide better extrapolation in high-slip, out-of-distribution regions where data is scarce [32]. Other nonlinear models, like XGBoost, LightGBM, and Random Forest could prove to be suitable alternatives. However, the potential marginal predictive improvements are not the focus of our research. Together, the Lasso and CatBoost models form the foundation for the ensemble approach proposed in this study.

CatBoost is recognized for its strong out-of-the-box performance with default hyperparameters [31]. After validation, the selected hyperparameters were a learning rate of 0.07 and a tree depth of 6. An early stopping criterion was also applied to prevent overfitting, terminating training when the validation loss reached its minimum.

For the linear models, sample weights were introduced, assigning a weight to each sample based on the absolute value of the slip. Internally, these weights are multiplied by the residuals in the loss function. Because high-slip regions are the most detrimental, this weighting emphasizes these regions, with the goal of improving model accuracy where it is most critical. Sample weights were not applied to the CatBoost models because they were designated as the steady-state predictors for the low-acceleration regime of the eventual ensemble. By maintaining a standard unweighted loss function for this component, the CatBoost models remained optimized for baseline accuracy without being biased toward the transient outliers that the linear model was specifically assigned to handle. This distinction ensures that the ensemble leverages the strengths of both architectures: the linear model provides robust extrapolation for high-slip ramps, while the gradient-boosted trees provide a stable, precise estimate for steady-state operation.

To assess generalization to unseen slip magnitudes associated with high web speeds that are absent from the training and validation data, a consistent experimental setup was applied across all data splits. In particular, the training and validation sets exclude the highest web speed of 60 m/min, which is reserved for the test set (see Table 2). A unified evaluation pipeline was established in which each model is assessed on both lower-speed and higher-speed operating regimes within each data split.

The distinction between lower-speed and higher-speed data is defined using two complementary segmentation strategies. The first, denoted as speed-based, partitions the data according to the imposed web speed, with runs exceeding 50 m/min classified as higher-speed regime. The second, denoted as quantile-based, identifies higher-speed operation using an upper quantile threshold of the traction roller acceleration distribution. This typically results in a smaller subset of samples corresponding to more extreme operating conditions.

The final ensemble model, illustrated in the rightmost block of Figure 3, combines the predictions of the linear and CatBoost models through a rule-based decision node. At each time step, the selection of the model is determined by a threshold defined on the upper quantile of the traction roller acceleration. If the acceleration exceeds this threshold, the prediction from the linear model is used. If this is not the case, the CatBoost prediction is selected. In this study, the highest 2.5% quantile of the traction roller acceleration distribution is defined as the high-speed regime for model selection. This exact selection is based on investigating the acceleration profiles of the training set to avoid data leakage. The threshold was set to include the two most elevated acceleration profiles of this training set. Both base models are trained on the same selected feature set, while the ensemble logic determines which prediction is propagated as the final output.

## 5. Results

This section evaluates the methodology discussed in Section 4, beginning with the feature selection results, to identify the key physical drivers of slip. The performance of the individual CatBoost and Lasso models is then assessed, followed by a quantitative and visual evaluation of the final ensemble model’s performance during both steady-state and transient operational regimes.

Table A1 and Table A2 illustrate the progression of the model error during the greedy forward selection process for the linear and CatBoost models, respectively. Rather than providing static importance weights, these scores track the optimization of the MSE as the feature subset expands. The most significant error reductions originate from features related to traction speed deltas and load cell fluctuations, confirming that the high-frequency dynamics of the winding process are the primary drivers of slip. As additional features, such as motor positions and setpoints, and longer statistical windows are incorporated, the MSE continues to decrease. However, this is with the expected diminishing returns as the primary variance in the dataset is captured by the initial highest ranked features.

A union of the features selected by both the linear and CatBoost models was used to train each model, enabling an objective comparison between them. The results of the speed- and quantile-based evaluations across all splits for the linear model are presented in Table 3. Similarly, the corresponding evaluations for the CatBoost model are shown in Table 4. These tables provide valuable insights into the models’ ability to extrapolate to unseen configurations. One notable observation is the difference in sample sizes when comparing the speed- and quantile-based evaluations. Specifically, the quantile-based evaluations contain significantly fewer samples in the high-speed segments, focusing primarily on the highest, critical slip regions.

The speed-based and quantile-based evaluations can only be compared in isolation, as the sample sizes for each subset differ between the two methods. Based on the results in Table 3 and Table 4, clear performance differences emerge between low-slip and high-slip operating regimes. In low-speed regimes, the CatBoost model consistently outperforms the linear model, achieving substantially lower prediction errors across training, validation, and test sets (e.g., validation score of 0.0550 versus 0.0949 in the speed-based low-speed split). This demonstrates CatBoost’s ability to capture nonlinear relationships when sufficient nominal data is available.

Alternatively, in high-speed and high-acceleration regimes, the linear model exhibits better prediction performance. Notably, for the unseen web speed of 60 m/min in the test set, the linear model achieves a substantially lower error than CatBoost (1.8740 versus 5.0188 in the speed-based split), and also a lower error in the quantile-based high-speed regime (8.5814 versus 26.4199). These results indicate that while CatBoost excels in nominal operating conditions, its predictions degrade rapidly under out-of-distribution slip levels, whereas the linear model provides more reliable extrapolation. This complementary behavior motivates the proposed ensemble strategy in which CatBoost is used for low-slip prediction and the linear model is employed to handle high-slip regimes.

Table 5 displays the speed-based and quantile-based evaluation results trained on the ensemble model. The ensemble reduces the CatBoost error at 60 m/min by approximately 54% in the speed-based evaluation (5.0188 to 2.3104) and by approximately 67.5% in the quantile-based evaluation (26.4199 to 8.5814) while maintaining comparable performance in low-speed regimes. These results demonstrate that the ensemble improves robustness and extrapolation in extreme operating conditions without sacrificing nominal performance.

**Figure 5:**
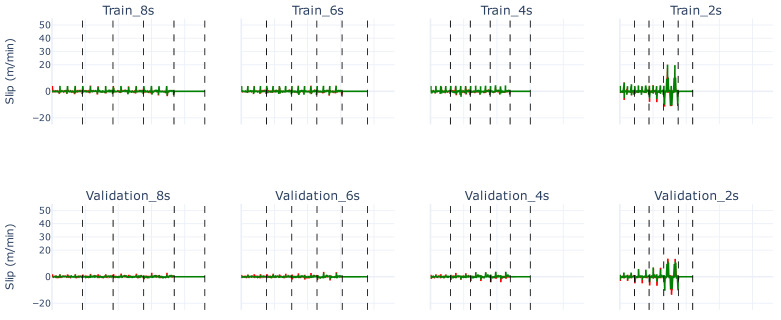

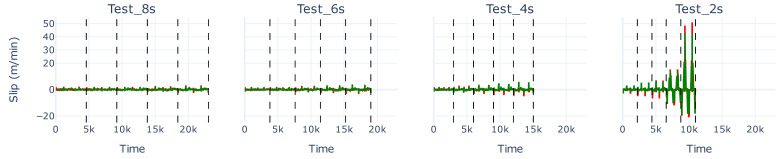
The various operational configurations of our use case, similar to Figure 2, with the ground truth slip label values illustrated in red and the predicted slip labels of the final ensemble model depicted in green. The training, validation, and test sets are shown from the top to bottom row respectively. The ramp time gets progressively shortened from left to right for each set. The vertical dashed lines each show the absolute value of the distinct web speeds found in Table 2.

This ensemble model enables an effective slip measurement, as illustrated in Figure 5. Similar to Figure 2, web speeds are separated and indicated with vertical dashed lines, corresponding to the increments presented in Table 2.

**Figure 6:**
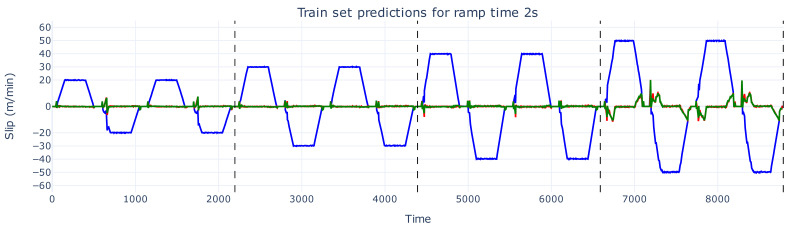
The slip prediction versus slip ground truth of the training set with a ramp time of 2 s. The actual slip values are shown in red, the prediction of these slip values are depicted in green, and the web speed is illustrated in blue.

**Figure 7:**
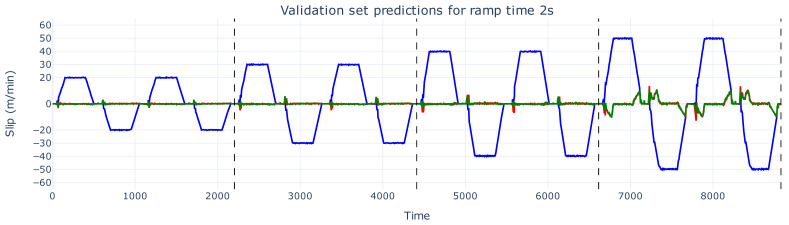
The slip prediction versus slip ground truth of the validation set with a ramp time of 2 s. The actual slip values are shown in red, the prediction of these slip values are depicted in green, and the web speed is illustrated in blue.

**Figure 8:**
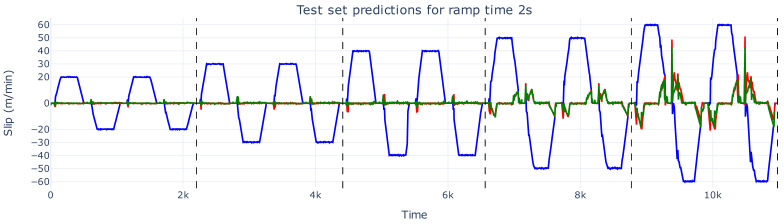
The slip prediction versus slip ground truth of the test set with a ramp time of 2 s. The actual slip values are shown in red, the prediction of these slip values are depicted in green, and the web speed is illustrated in blue.

Figure 6, Figure 7 and Figure 8 show the predicted slip values for the training, validation, and test sets, respectively, for a ramp time of 2 s, zoomed in from Figure 5. In these figures, the actual slip ground truth slip values are shown in red, the predicted slip values in green, and the real web speed in blue. The predictions for the training and validation sets (Figure 6 and Figure 7) are generally accurate, with only minor spikes not captured correctly.

The most notable results are observed in the test set (Figure 8). In particular, when the web speed reaches 60 m/min, the predictions deviate once the slip values enter out-of-distribution regions. For web speeds within the training distribution (up to 50 m/min), the predictions remain reasonably accurate. Even at 60 m/min, the predicted slip values are higher than those seen in the training and validation sets, indicating that the linear model is extrapolating to unseen ground truth values, although not sufficiently to fully capture the extreme slip.

## 6. Discussion

The evaluation results indicate that the proposed ensemble model generalizes well across operating conditions. While CatBoost effectively captures low-slip behavior, the linear model provides robust predictions in high-slip, out-of-distribution regions. Feature selection confirms that the instantaneous speed mismatch between traction rolls, captured by the high-frequency traction delta, is the most informative predictor. This validates the physical intuition that asynchronicity between rollers drives slip. By weighting these high-slip regions in the linear model, the framework emphasizes the most critical operational conditions, which is essential to prevent material damage.

Despite these strengths, several limitations remain. The experimental setup represents only a portion of a full-scale industrial web winding line, and real-world conditions could introduce additional noise from environmental factors or multiple processing stations. The current dataset includes a single web material, limiting direct generalization to materials with different mechanical properties. While we believe that the proposed approach is applicable to other materials with similar material specifications, materials with substantially different mechanical or surface characteristics remain to be systematically evaluated in future work.

A key consideration in this study is that slip measurements were localized to Encoder Roll 1. In complex winding systems, slip is a dynamic phenomenon that can propagate or vary across different mechanical components due to localized tension gradients and surface variations. While the current framework accurately characterizes slip at this specific point, the absolute values may differ in other sections of a production machine. Consequently, while the methodology is robust, the specific convergence of the model may require recalibration when applied to different mechanical architectures or use cases where slip propagation is more pronounced.

Regarding the inclusion of forward temporal shifts (lags), and the associated temporal look-ahead, the prediction window is mathematically bound by the tsflex window sizes and specific feature shifts. In this implementation, these shifts are relatively small. For instance, a shift of 2 samples at a 50 Hz sampling rate is equivalent to 40 ms. To put this in perspective, even under extreme conditions where slip reaches 50 m/min, this 40 ms window corresponds to a physical material length of only approximately 33.3 mm. Given that instantaneous mechanical intervention is physically impossible within such a narrow timeframe due to actuator latency and system inertia, these forward shifts were incorporated to enhance the model’s predictive performance and better characterize the onset of slip.

It is important to distinguish between the offline modeling phase and the online prediction phase in this research. The models are trained offline using historical data collected with a temporary encoder. Next, the trained models are used for online inference on real-time machine signals without further model parameter updates. This enables continuous, real-time slip monitoring at the machine’s sampling rate. Furthermore, the computational simplicity of the selected feature subset, as reflected in Table A1 and Table A2, further supports the suitability of the pipeline for real-time deployment.

As noted in Section 2, validating our methodology on other similar datasets is challenging as no publicly available datasets of this type exist. This highlights the advantage of opening our dataset so it can be used for future research and benchmarking. Additionally, the generic approach of using a boosting algorithm for low-value ground truth regions and a linear model for higher, out-of-distribution values could be applied to other datasets. For example, predicting Remaining Useful Life (RUL) in faulty bearing datasets using accelerometer data might exhibit a comparable imbalance between nominal operating conditions and rare, extreme degradation states [33]. Validating the methodology for other use cases is, however, beyond the scope of the present study.

Future work should focus on scaling the approach to full industrial machines, investigating a broader range of web materials, incorporating realistic environmental noise, and exploring other fault types in continuous web processes. These efforts would further validate the methodology and strengthen its utility for industrial predictive maintenance and process optimization.

## 7. Conclusions

Although slip can be measured directly by installing high-resolution encoders that monitor true web speed, industrial R2R machines rarely include such instrumentation. The cost, complexity of the cabling, and machine downtime required for installation make high-resolution sensing impractical for widespread deployment or retrofitting. Instead, most installed sensors are low-cost, low-resolution devices that track cumulative web length rather than instantaneous speed, rendering them unable to detect slip during short high-acceleration events.

This study presents a data-driven framework for predicting slip in 2D web winding machines without the need for dedicated encoder sensors. By leveraging existing machine signals combined with feature engineering and an ensemble of linear and CatBoost models, slip can be estimated across a range of operating conditions. The ensemble approach allows linear models to provide reliable extrapolation in out-of-distribution high-slip regions, while gradient boosted models capture complex patterns in low-slip regions within the distribution of the training data.

The methodology enables operators to investigate critical slip events, potentially improving product quality, reducing material waste, and allowing safer high-speed operation. In general, the study demonstrates that virtual sensors, supported by careful feature engineering and ensemble modeling, offer a practical and cost-effective solution for slip monitoring in industrial web winding machines.

To further encourage innovation and promote comparative analysis in predictive maintenance, we are making our complete experimental dataset publicly available. By providing our benchmarks and code repository, we invite the research community to build upon these findings, evaluate alternative predictive architectures, and further advance the state-of-the-art in virtual sensing for web processing machines.

Future work should focus on evaluating the generalizability of the approach across a broader range of materials, machine configurations, and operating conditions. We invite the research community to build upon the publicly available dataset and code repository to advance this direction.

## Figures and Tables

**Table 1:** Physical properties and specifications of the Black Kerdi polyethylene membrane used as the experimental web material.

	Black Kerdi
Width (cm)	55
Thickness (µm)	200
Mass density (g/m^2^)	150
Structure	three layers
Est. stiffness (N/m)	5500
Est. damping (N/m/s)	350

**Table 3:** Speed- and quantile-based evaluation for the linear model trained on all selected features. The low speed for the speed-based evaluation signifies an imposed speed that is smaller than 50 while the high speed is an imposed speed equal to 50, except for the test set where the explicit high speeds are listed. The quantile-based evaluation considers the highest 2.5% quantile of the traction roll acceleration as high speed.

	Speed-Based				Quantile-Based			
	Low Speed		High Speed		Low Speed		High Speed	
	Score	Samples	Score	Samples	Score	Samples	Score	Samples
Train	0.1076	40,660	0.2241	13,578	0.1212	52,879	0.7431	1359
Validation	0.0949	40,764	0.2757	13,620	0.0974	53,007	1.7846	1377
Test	0.0988	40,621	0.2804 (50$\frac{m}{\min}$) 1.8740 (60$\frac{m}{\min}$)	13,636 (50$\frac{m}{\min}$) 13,578 (60$\frac{m}{\min}$)	0.2371	65,774	8.5814	2061

**Table 4:** Speed- and quantile-based evaluation for the CatBoost model trained on all selected features. The low speed for the speed-based evaluation signifies an imposed speed that is smaller than 50, while the high speed is an imposed speed equal to 50, except for the test set where the explicit high speeds are listed. The quantile-based evaluation considers the highest 2.5% quantile of the traction roll acceleration as high speed.

	Speed-Based				Quantile-Based			
	Low Speed		High Speed		Low Speed		High Speed	
	Score	Samples	Score	Samples	Score	Samples	Score	Samples
Train	0.0169	40,660	0.0284	13,578	0.0184	52,879	0.0732	1359
Validation	0.0550	40,764	0.1811	13,620	0.0479	53,007	1.5755	1377
Test	0.0605	40,621	0.2116 (50$\frac{m}{\min}$) 5.0188 (60$\frac{m}{\min}$)	13,636 (50$\frac{m}{\min}$) 13,578 (60$\frac{m}{\min}$)	0.2894	65,774	26.4199	2061

**Table 5:** Speed-based and quantile-based evaluation for the ensemble model trained on all selected features with the highest 2.5% quantile of the traction roll acceleration considered as high speed.

	Speed-Based				Quantile-Based			
	Low Speed		High Speed		Low Speed		High Speed	
	Score	Samples	Score	Samples	Score	Samples	Score	Samples
Train	0.0220	40,660	0.0802	13,578	0.0184	52,879	0.7431	1359
Validation	0.0604	40,764	0.1859	13,620	0.0479	53,007	1.7846	1377
Test	0.0643	40,621	0.2009 (50$\frac{m}{\min}$) 2.3104 (60$\frac{m}{\min}$)	13,636 (50$\frac{m}{\min}$) 13,578 (60$\frac{m}{\min}$)	0.2894	65,774	8.5814	2061

## Data Availability

Data used within this research project is made publicly available (see https://github.com/predict-idlab (accessed on 27 February 2026)) to stimulate setting benchmarks. We would like to thank Jasper De Viaene for the data acquisition. The code written for this research project is transformed into open-source Jupyter Notebooks and can be found at https://github.com/predict-idlab (accessed on 27 February 2026).

## Appendix A

**Table A1:** Evolution of the model error during greedy forward selection performed with linear models. The *Score* column represents the Mean Square Error (MSE) of the model using all features from the top to the current row. The decreasing scores illustrate the marginal performance gain provided by each additional sensor or temporal transformation.

Feature Source	Operation	Window (*w*)	Shift	Score
Δ Traction Speed	Last	2	Forward 2	0.2775
LoadCell2 Diff	Mean	4	Forward 1	0.1490
LoadCell2 Diff	Mean	32	None	0.1415
LoadCell1 Diff	Mean	4	None	0.1402
Δ Traction Speed	Mean	64	None	0.1401
LoadCell2 Diff	Mean	8	Forward 2	0.1401
LoadCell1 Diff	First	2	Forward 1	0.1401
LoadCell2 Diff	Std Dev	16	None	0.1401
LoadCell1 Diff	Diff	2	Forward 1	0.1401

**Table A2:** Evolution of the model error during greedy forward selection performed with CatBoost models. The *Score* column represents the Mean Square Error (MSE) of the model using all features from the top to the current row. The decreasing scores illustrate the marginal performance gain provided by each additional sensor or temporal transformation.

Feature Source	Operation	Window (*w*)	Shift	Score
Δ Traction Speed	Last	2	Forward 2	0.2360
LoadCell2 Diff	Mean	4	Forward 1	0.1441
Traction2 Position	Diff	2	Backward 2	0.1157
Traction1 Setpoint	Diff	2	Backward 2	0.1079
LoadCell1 Diff	Mean	16	None	0.1008
LoadCell1 Diff	Mean	4	Forward 1	0.0962
LoadCell2 Diff	Std Dev	64	None	0.0945

**Table A3:** Specifications of sensors and actuators of the use case.

	Description	Function	Type	Supplier	Output	Accuracy
Wind roll	Optical distance sensor with laser light	Roll radius	O1D100	IFM Electronic N.V.	Analog	±15 mm
Load cell	Load cell	Web tenstion	PD2125	ERHARDT + LEIMER	Analog	0.2%
Dancer	Inductive inclinometer	Position dancer	B2N60H	TURCK GmbH	Analog	0.14°
Accumulator	Optical distance sensor with laser light	Position accumulator	O1D100	IFM Electronic N.V.	Analog	±15 mm
Edge sensor	Photoelectric sensor	Position web edge	QS18	MULTIPRO X N.V.	Digital	/

**Table A4:** Specifications of the motors of the use case.

	Motor Type	Drive Type	Power (W)	Torque (Nm)	Gear	Gear Ratio	Brake	Sensor
Wind roll	Induction	S120	1500	75	Bevel	6.51	Yes	Incr. Enc. (1024TTL)
Traction roll	PMSM	S210	1000	3.2	Bevel	9.35	No	Abs. Enc. (22bit)
Dancer	PMSM	S210	400	1.27	Helican	142.23	Yes	Abs. Enc. (22bit)
Accumulator	PMSM	S210	940	3.6	Bevel	32.78	Yes	Abs. Enc. (22bit)
Edge control	PMSM	IC	60	750 N	/	/	No	Potentiometer
